# Effectiveness of Artificial Intelligence in Screening Esophagogastroduodenoscopy

**DOI:** 10.7759/cureus.79935

**Published:** 2025-03-02

**Authors:** Kyoichi Adachi, Yuri Ebisutani, Yuko Matsubara, Eiko Okimoto, Norihisa Ishimura, Shunji Ishihara

**Affiliations:** 1 Health Center, Shimane Environment and Health Public Corporation, Matsue, JPN; 2 Second Department of Internal Medicine, Faculty of Medicine, Shimane University, Izumo, JPN

**Keywords:** ai, endoscopy, esophageal cancer, gastric cancer, screening

## Abstract

Background: This retrospective study investigated the usefulness of the EW10-EG01 artificial intelligence (AI) application for screening esophagogastroduodenoscopy (EGD).

Methodology: A total of 7,655 subjects (4,863 men, 2,792 women; mean age 54.9±10.1 years) who underwent EGD during a medical checkup were enrolled in the study. The number of diagnosed upper gastrointestinal tumors was compared between EGD examinations performed with and without the AI system.

Results: EGD examinations with and without the AI system were performed on 3,841 and 3,814 subjects, respectively. Biopsy procedures were more frequently performed, and examination time was longer in EGD with AI applications than in those without. Upper gastrointestinal tumors diagnosed by EGD with and without AI were 39 (1.02%) and 24 (0.63%), respectively (*P* = 0.062). There was a significant difference in the detection rate of esophageal and gastric tumors between EGD with (30, 0.78%) and without (14, 0.37%) the AI system (*P *= 0.017). When endoscopists were divided into three groups based on their experience with the EW10-EG01 application, higher detection rates of esophageal and gastric tumors were observed in each group when using EGD with AI.

Conclusions: Usage of the EW10-EG01 AI system may be useful for screening EGD due to the increased esophageal and gastric tumor detection rate.

## Introduction

Esophagogastroduodenoscopy (EGD) examinations are widely performed for the detection of various types of upper gastrointestinal tumorous lesions. However, tumor detection in an early stage can be difficult, especially for endoscopists with limited clinical experience [1-3]; thus, several different diagnostic methods, including narrow band imaging (NBI), linked color imaging (LCI), blue laser imaging (BLI), and magnified endoscopy, are often used to improve the diagnostic accuracy of an EGD examination. Recently, artificial intelligence (AI) methods have been produced, and the efficacy of EGD with the use of an AI application using both still images and video clips has been demonstrated in several studies [4-10]. EGD with an AI application is thus considered to have an important role for not only better detection rate but also accurate diagnosis of tumors noted in the upper gastrointestinal tract. The EW10-EG01 AI system (FUJIFILM, Tokyo, Japan) was developed to improve the detection rate of esophageal and gastric tumors during an EGD examination, with approval for use for pharmaceutical clinical practice in Japan received in 2022. This AI application is considered to be useful for EGD screening examinations, such as population-based examinations for gastric cancer, as those are sometimes performed by non-experienced endoscopists. However, whether the use of the EW10-EG01 AI application improves the detection rate of esophageal and gastric tumors in screening EGD examinations remains to be clarified. This retrospective study was performed to investigate the usefulness of the EW10-EG01 AI application for EGD performed as part of an annual checkup.

## Materials and methods

**Figure 1:**
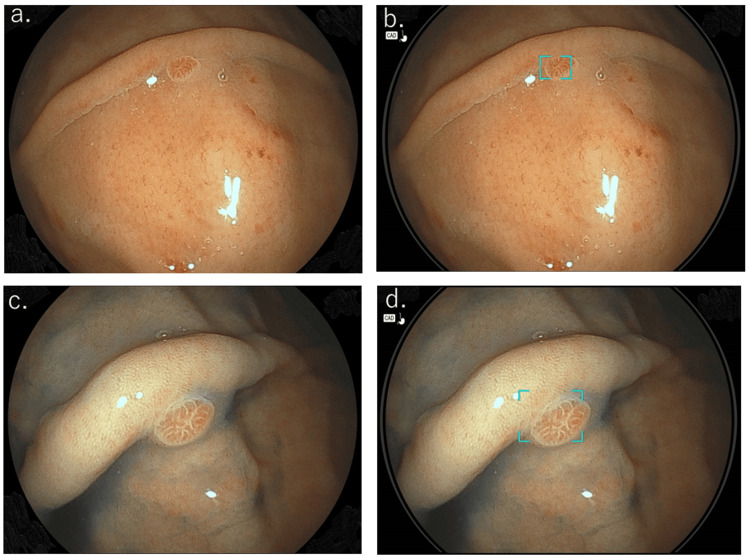
Endoscopic image of a raspberry-type, gastric foveolar-type tumor. Representative endoscopic image of the greater curvature of the upper body fornix, showing a 3 mm reddish, raspberry-like lesion. Histological findings from the biopsy revealed a foveolar-type adenoma tumor (a, b: white light imaging; c, d: imaging after indigo carmine spraying). Real-time images obtained using the EW10-EG01 AI application during EGD (b and d) indicated a possible tumor, highlighted by a square.

**Figure 2:**
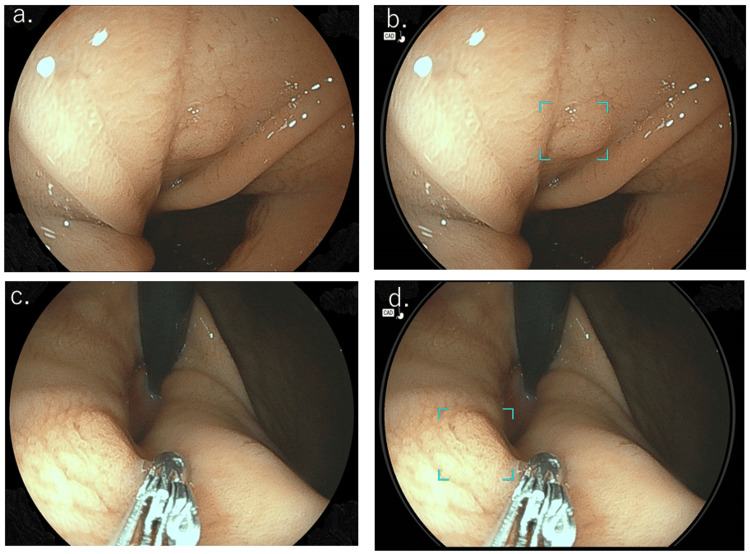
Endoscopic image of fundic-gland-type gastric adenocarcinoma. Representative endoscopic image of the cardiac portion showing a 5 mm flat, elevated, submucosal tumor-like lesion. Histological findings from endoscopic mucosal resection revealed submucosal invasion. Real-time images using the EW10-EG01 AI application during EGD (b and d) indicated a possible tumor, highlighted by a square.

The present subjects were selected from individuals who underwent an EGD examination at the Shimane Environment and Health Public Corporation Health Center as part of a medical checkup between January 2023 and June 2024, as the EW10-EG01 AI application was introduced in mid-December 2022 into one of the two endoscopic systems (LASEREO 7000 system, FUJIFILM, Tokyo, Japan) used at our institute. During an EGD examination, this AI application system can automatically indicate possible esophageal and gastric tumors within the viewing site (Figures 1, 2). On the other hand, routine observations of the stomach and duodenum are done with white light imaging (WLI), and then observations using LCI are performed when the endoscopist notes an abnormal finding, including gastric mucosal atrophy (GMA) cases. Several investigators have found that a gastric epithelial tumor can be easily detected by endoscopic observation with LCI, based on enhanced color differences in areas shown as red [11,12]. The EW10-EG01 AI application is designed to detect esophageal tumors during endoscopic observations using LCI and BLI and to identify gastric tumors when using WLI and LCI. The EW10-EG01 AI application was not developed for the detection of duodenal tumors. However, the application remains on during endoscopic observation of the duodenum, and duodenal tumor-like lesions are sometimes highlighted. Thus, the AI application system is generally functional during endoscopic observations for esophageal tumors using BLI and for gastric tumors using WLI during routine EGD examination in our institute. The EW10-EG01 AI system can detect changes in color tone as well as irregularities in the esophageal and gastric walls during an EGD examination. Non-tumorous lesions, such as benign polyps and erosions, are frequently detected by the EW10-EG01 AI system, although no known studies of its sensitivity and/or specificity for esophageal and gastric tumor detection have been presented. As a result, when the AI application detects a lesion, a judgment must be made to determine whether it is truly a tumorous lesion requiring treatment.

The total number of patients who underwent EGD during the study period was 7,706, of whom 35 with a history of gastrectomy were excluded from the analysis. During the study period, eight different endoscopists performed EGD examinations, with two on duty each day. The daily schedule was planned in advance to designate which endoscopist would perform EGD examinations using the AI-equipped system. Each day, patients were randomly allocated to one of the two endoscopists and EGD systems in sequential order based on their arrival time at the endoscopy room.

All EGD examinations were performed by experienced endoscopists using an EG-L580NW endoscope (FUJIFILM). Eight endoscopists (A-H) performed these examinations during the study period. A, B, C, D, and E were full-time workers at our institute. A and B were employed throughout the study period, and C, D, and E were transferred to or from another hospital during that time. On the other hand, F, G, and H performed EGD examinations at our institute during the study period on a part-time basis. A, B, F, G, and H were certified by the Japan Society of Gastroenterological Endoscopy (JGES), while C, D, and E each had at least five years of experience performing EGD examinations at the beginning of the study period. The numbers of EGD examinations of the present subjects performed by endoscopists A, B, C, D, E, F, G, and H were 2,608, 2,256, 1,721, 298, 257, 515, 8, and 8, respectively. For the present study, data obtained from 7,655 patients (4,863 men, 2,792 women; mean age 54.9 ± 10.1 years) were analyzed, after excluding 16 who underwent EGD examinations by endoscopist G or H. Number of biopsy procedures, EGD examination time, and tumor detection rate were analyzed after dividing the six endoscopists into certified (A, B, F) and non-certified (C, D, E). The analysis of examination time was conducted using the results of 100 consecutive EGD examinations performed by certified and non-certified endoscopists and without a biopsy procedure during the middle portion of the study period.

*Helicobacter pylori* infection status was comprehensively determined as positive, post-eradication, or uninfected, based on a detailed medical history obtained during an interview conducted by a public health nurse, as well as diagnostic testing for *H. pylori* infection and EGD results. During the study period, serum anti-*H. pylori *IgG antibody assays for determining *H. pylori* infection were performed using a SphereLight H. pylori antibody J® kit or L-type Wako H. pylori antibody J® kit (FUJIFILM Wako Pure Chemical Corporation, Osaka, Japan). Subjects who had undergone therapy without successful eradication were not included in the post-eradication group. If the success of eradication therapy could not be confirmed, patients were advised to undergo an *H. pylori *stool antigen test at our hospital.

Degree of endoscopic GMA and endoscopic findings indicating positive for *H. pylori* infection, such as nodular gastritis, spotty and/or diffuse redness of fundic gland mucosa, and sticky mucous [13-15], were carefully examined in each case. Using the Kimura-Takemoto classification, the degree of GMA was classified into six grades: C1, C2, C3, O1, O2, and O3 [16]. Patients were classified as *H. pylori*-uninfected when there was no medical history of eradication therapy and no positive findings for *H. pylori* infection, and the endoscopic GMA determination was no atrophy or C1, as well as negative for *H. pylori* testing when that was performed. A patient was determined to be positive for *H. pylori *infection if test results were positive, including endoscopic GMA (C2-O3) and/or endoscopic findings indicative of *H. pylori *infection. Subjects with endoscopic GMA (C2-O3) and negative results in *H. pylori* testing were assigned to the *H. pylori*-eradicated group. As a result, the 7,655 subjects in this study were divided into the *H. pylori*-positive (*n* = 211), *H. pylori*-eradicated (*n* = 2,537), and *H. pylori*-uninfected (*n* = 4,907) groups. For data analysis, *H. pylori*-positive and *H. pylori*-eradicated cases were combined into a single *H. pylori*-positive group (*n* = 2,748).

EGD examination results with and without the AI system were compared based on the number of cases diagnosed with a tumor in the esophagus, stomach, or duodenum during the study period. Esophageal cancer lesions, gastric tumors (including gastric epithelial tumors and lymphomas), and duodenal tumors were analyzed in this study. Duodenal tumors included cancer, adenoma, and lymphoma, although duodenal tumors are not a target lesion of the EW10-EG01 AI application. All tumors analyzed in this study were histologically diagnosed based on specimens obtained through endoscopic biopsy, endoscopic submucosal resection, and/or surgical treatment. Upper gastrointestinal polypoid lesions, such as esophageal papilloma, gastric fundic gland polyps, and hyperplastic polyps, were not included in the analysis, as treatment for these lesions is generally considered unnecessary. In this study, raspberry-type gastric foveolar-type tumors were included as gastric tumors, although recent reports have suggested that this type of tumor should be classified as foveolar-type adenoma [17,18].

Submucosal tumors were also excluded from the analysis in this study, as all detected during the study period were determined not to require treatment. Because the study was conducted retrospectively, it was unknown whether the AI application initially identified a diagnosed upper gastrointestinal tumor.

Statistical analyses were performed using the chi-squared test, Fisher's exact test, Mann-Whitney U test, and Kruskal-Wallis test. All calculations were conducted using StatView software, version 5.0 (Abacus Concepts Inc., Berkeley, CA), with a *P*-value < 0.05 considered statistically significant.

This study was conducted in accordance with the Declaration of Helsinki, and the protocol was approved by the Ethics Committee of the Shimane Environment and Health Public Corporation (201703-202401). Written informed consent was obtained from each enrolled subject before the medical checkups, indicating that clinical data could be used for research without the release of personal information. Additionally, subjects were given the option to withdraw from this retrospective study at any time by notifying the study manager.

## Results

During the study period, 3,841 EGD examinations were performed with the EW10-EG01 AI application and 3,814 without it. Gender, age, *H. pylori* infection status, and endoscopist group did not differ significantly between the EGD with AI and EGD without AI groups (Table 1).

**Table 1:** Characteristics of study subjects. *Positive group: *H. pylori*-positive and post-eradicated cases. **Certified by the Japan Society of Gastroenterological Endoscopy (JGES). EGD, esophagogastroduodenoscopy; AI, artificial intelligence; SD, standard deviation

	Endoscopic system		*P*-value
	With AI	Without AI	
Number of EGD examinations	3,841	3,814	
Male/female	2,430/1,411	2,433/1,381	0.229
Age (years), mean ± SD	55.0 ± 10.2	54.7 ± 10.1	0.136
*Helicobacter pylori* infection status			0.435
Positive group*	1,365 (35.5%)	1,383 (36.3%)	-
Uninfected	2,476 (64.5%)	2,431 (63.7%)	-
Number of EGD examinations performed			0.509
Certified endoscopists**	2,615 (68.1%)	2,764 (72.5%)	
Non-certified endoscopists	1,226 (31.9%)	1,050 (27.5%)	

When analyzing the number of EGD procedures with biopsies for histological diagnosis, gastric biopsies were performed more frequently during EGDs with AI compared to those without AI. The number of esophageal biopsy procedures in EGDs with AI tended to be higher in EGDs with AI than those without. On the other hand, the number of duodenal biopsy procedures was not different between EGDs with and without AI.

When non-certified endoscopists performed the examination, the time required for EGD with the AI application was significantly longer as compared to without its use (Table 2).

**Table 2:** Number of biopsy procedures and EGD examination time. *Certified by the Japan Society of Gastroenterological Endoscopy (JGES). **A total of 100 consecutive EGD examinations with and without AI application were performed by certified and non-certified endoscopists. Those with a biopsy procedure were not included. AI, artificial intelligence; EGD, esophagogastroduodenoscopy

	Endoscopic system		*P*-value
	With AI	Without AI	
EGD with the biopsy procedure			
Total	240 (6.2%)	181 (4.8%)	0.004
Certified endoscopists*	153 (5.8%)	130 (4.7%)	0.060
Non-certified endoscopists	87 (7.1%)	51 (4.9%)	0.025
Biopsy site			
Esophagus	17 (0.4%)	8 (0.2%)	0.074
Stomach	205 (5.3%)	153 (4.0%)	0.006
Duodenum	18 (0.5%)	20 (0.5%)	0.729
EGD examination time (seconds)**			
Total	311 ± 52	291 ± 51	<0.001
Certified endoscopists*	304 ± 45	302 ± 55	0.403
Non-certified endoscopists	318 ± 59	281 ± 45	<0.001

Among all subjects, 63 were diagnosed with an upper gastrointestinal tumor during the study period. Two cases were gastric adenocarcinomas of the fundic-gland type, which showed slight submucosal invasion in histological findings following endoscopic submucosal resection. Other gastric, esophageal, and duodenal cancers were diagnosed as mucosal cancers based on histological findings after endoscopic submucosal resection. Therefore, none of the subjects with upper gastrointestinal cancer detected during the study period required chemotherapy, radiation, esophagectomy, or gastrectomy.

The number of upper gastrointestinal tumors, including duodenal tumors, diagnosed by EGD with AI and EGD without AI was 39 (1.02%) and 24 (0.63%), respectively (*P* = 0.062). Four patients were diagnosed with esophageal cancer using EGD with AI, compared to two patients diagnosed without AI (*P* = 0.419). For gastric tumors, the number diagnosed using EGD with AI was significantly higher (*P* = 0.024). Additionally, the AI-assisted findings tended to lead to a more frequent diagnosis of raspberry-type gastric foveolar-type tumors (*P* = 0.077). In addition, flat and depressed esophageal cancer and gastric well-differentiated adenocarcinomas were diagnosed more frequently based on EGD findings obtained with the AI application (*P* = 0.006). There was a significant difference in the detection rate of esophageal and gastric tumors between EGD with AI and EGD without AI (30, 0.78%, vs. 14, 0.37%, *P* = 0.017). On the other hand, there was no difference in the number of diagnosed duodenal tumors between EGD examinations performed with and without the AI system. Findings from analyses conducted after dividing cases by endoscopist classification showed that esophageal and gastric tumors were diagnosed more frequently when a certified endoscopist performed EGD with the AI system (*P* = 0.018). In contrast, there was no significant difference in the detection rate for EGD examinations performed by non-certified endoscopists (*P* = 0.440) (Table 3). 

**Table 3:** Diagnosed upper gastrointestinal tumors. *Certified by the Japan Society of Gastroenterological Endoscopy (JGES). MALT, mucosa-associated lymphoid tissue

	Endoscopic system		*P*-value
	With AI	Without AI	
Esophageal tumor	4 (0.10%)	2 (0.05%)	0.419
Esophageal cancer	4	2	
Elevated/flat and depressed type	(0/4)	(0/2)	
Gastric tumor	26 (0.68%)	12 (0.32%)	0.024
Raspberry-type gastric foveolar-type tumor	12	4	
Gastric adenocarcinoma of the fundic-gland type	5	6	-
Well-differentiated adenocarcinoma	6	1	-
Elevated/flat and depressed type	(2/4)	(0/1)	
Foveolar-type adenocarcinoma	2	0	-
MALT lymphoma	1	1	
Esophageal and gastric tumors (total)	30 (0.78%)	14 (0.37%)	0.017
Certified endoscopists*	24 (0.92%)	11 (0.40%)	0.018
Non-certified endoscopists	6 (0.49%)	3 (0.29%)	0.440
Duodenal tumors	9 (0.23%)	10 (0.26%)	0.806
Well-differentiated adenocarcinoma	2	1	
Adenoma	6	7	
Lymphoma	1	2	
Upper gastrointestinal tumors (total)	39 (1.02%)	24 (0.63%)	0.062

Additionally, gastric tumors tended to be diagnosed more frequently based on EGD findings obtained with the AI application compared to without it in both *H. pylori*-positive (0.66% vs. 0.22%, *P* = 0.079) and *H. pylori*-uninfected (0.69% vs. 0.37%, *P* = 0.127) cases (Table 4).

**Table 4:** Diagnosed gastric tumors in subjects grouped by Helicobacter pylori status. *H. pylori-positive and post-eradicated cases.

	Endoscopic system		*P*-value
	With AI	Without AI	
Status of *H. pylori* infection			
Positive group*	9 (0.66%)	3 (0.22%)	0.079
Uninfected	17 (0.69%)	9 (0.37%)	0.127

## Discussion

AI application systems are increasingly being used in various medical fields because of the large possibility for improvement in the quality of care. For the development of image recognition systems, deep learning using various training images is useful and many studies have shown improvements in the quality of EGD examinations with the use of an AI application system [4-10]. The EW10-EG01 system was the first to be approved for EGD examinations performed in clinical practice in Japan, and this study was conducted to investigate its usefulness for individuals undergoing EGD as part of a medical checkup.

To the best of our knowledge, the present findings are the first to demonstrate that EGD with the EW10-EG01 system is highly effective for the detection of esophageal and gastric tumors as compared to examinations without the use of that AI program. In the present cohort, esophageal and gastric biopsy procedures were more frequently performed, and the examination time was longer for EGD with the AI application. The greater number of biopsy procedures in patients who underwent EGD with AI may have been because the use of that combination resulted in more frequent detection of lesions suspected to be a tumor, including non-tumorous lesions. In addition, the examination time for EGD with AI was longer compared to EGD without AI. That difference was considered to be caused by the extra time needed to judge whether a lesion detected by the AI application was truly tumorous. Notably, EGD performed by non-certified endoscopists had a significantly longer examination time, possibly due to the additional time required to evaluate detected lesions.

All subjects with esophageal and gastric cancer diagnosed during the study period were subsequently treated by endoscopic resection. These results indicate that the AI application is highly useful for the early detection of esophageal and gastric tumors during screening EGD examinations. On the other hand, the EW10-EG01 AI system was not designed for the detection of duodenal tumors. Indeed, the detection rate of duodenal tumors in this study did not differ between EGD examinations performed with and without the AI system.

The higher detection rates of esophageal and gastric tumors using EGD with the EW10-EG01 AI application in the present study were associated with the increased detection of esophageal cancer, gastric raspberry-type foveolar-type tumors, and well-differentiated adenocarcinomas. In addition, esophageal cancer and gastric well-differentiated adenocarcinomas diagnosed during the study period were mainly the flat and depressed type. On the other hand, a raspberry-type gastric foveolar-type tumor is shown by endoscopy as a polypoid lesion with a reddish fine granular surface [18,19]. Thus, EGD with the EW10-EG01 AI application was found to be effective in detecting not only elevated lesions but also flat and depressed-type lesions.

For the present cohort, gastric tumors tended to be diagnosed more frequently using EGD with AI compared to without AI in both the *H. pylori*-positive (including eradicated cases) and *H. pylori*-uninfected groups. Notably, the gastric tumor detection rate in uninfected patients who underwent an EGD examination with the EW10-EG01 AI system was 0.69%. The development of gastric cancer in *H. pylori*-uninfected individuals in Japan has recently been frequently reported [17-23]. We previously reported that the prevalence of gastric tumors in *H. pylori*-uninfected subjects was 0.28% during a period when nearly all EGD examinations were performed without the addition of an AI system [24]. Thus, the use of EGD with the EW10-EG01 AI system is considered to be effective for detecting a gastric tumor not only in *H. pylori*-uninfected patients but also in *H. pylori*-positive patients, although a larger scale study will be needed to confirm these findings.

Although several previous studies have noted that the use of an AI application is expected to improve the quality of EGD examinations performed by non-experienced endoscopists [4-10], the present findings did not indicate increased detection of esophageal or gastric tumors in such examinations with the EW10-EG01 AI system when performed by non-certified endoscopists. On the other hand, there was a significant difference when those were performed by certified endoscopists. It is, therefore, recommended that endoscopists who use this system should have a high level of ability for differential diagnosis of detected lesions. Further development of the EW10-EG01 AI application is also needed for easier and more accurate diagnosis of upper gastrointestinal tumors revealed by EGD, especially in examinations performed by non-experienced endoscopists. In addition, studies that analyze the usefulness of the EW10-EG01 AI application in comparison with other AI systems are needed.

This study has several limitations, including its retrospective design and performance at a single center. Also, the number of study subjects was relatively small, though 63 upper gastrointestinal tumors, including 44 esophageal and gastric tumors, were diagnosed during the study period. Our institution was established to perform medical checkups, and the age of the present subjects may be younger as compared to those who undergo EGD in clinical practice or as part of population-based screening. Nevertheless, the results obtained serve as a valuable reference for all types of EGD examinations conducted for the detection of esophageal and gastric tumors. Additional large-scale prospective studies are recommended to confirm the usefulness of the EW10-EG01 AI application.

## Conclusions

This retrospective study was performed to investigate the usefulness of the EW10-EG01 AI application for EGD screening examinations. The use of the EW10-EG01 AI system may be beneficial for screening EGD, as it increases the detection rate of esophageal and gastric tumors. On the other hand, examination time was longer, and biopsy procedures were more frequently performed in patients who underwent EGD with AI. Further development is needed for more efficient detection of tumorous lesions that require treatment.
